# Oral health, socio-economic and home environmental factors associated with general and oral-health related quality of life and convergent validity of two instruments

**DOI:** 10.1186/s12903-015-0009-7

**Published:** 2015-02-24

**Authors:** Janice S Paula, Marcelo C Meneghim, Antônio C Pereira, Fábio L Mialhe

**Affiliations:** Department of Community Dentistry, Division of Health Education and Health Promotion, Piracicaba Dental School, University of Campinas –UNICAMP, P.O. BOX 52, Piracicaba, SP 13414-903 Brazil

**Keywords:** Quality of life, Oral health, Children, AUQUEI, CPQ_11–14_

## Abstract

**Background:**

The objective of this study was to evaluate the convergent validity between the domains of the Autoquestionnaire Qualité de Vie Enfant image (AUQUEI) and the Child Perceptions Questionnaire instrument (CPQ_11–14_) among schoolchildren and to assess the difference between socio-economic and clinical variables associated with their scores.

**Methods:**

An analytical cross-sectional study was conducted in Juiz de Fora, Minas Gerais, Brazil, with 515 schoolchildren aged 12 years from 22 public and private schools, selected with the use of a random multistage sampling design. They were clinically examined for dental caries experience (DMFT and dmft index) and orthodontic treatments needs (DAI index) and were asked to complete the Brazilian versions of Child Perception Questionnaire (CPQ_11–14_) and Autoquestionnaire Qualité de Vie Enfant image (AUQUEI). In addition, a questionnaire was sent to their parents inquiring about their socio-economic status and home characteristics. The convergent validity of the Brazilian versions of CPQ_11–14_ and AUQUEI instruments was analyzed by Spearman’s correlation coefficients. For comparison between the summarized scores of each questionnaire with regard to the schoolchildren’s socio-environmental and clinical aspects the nonparametric Mann–Whitney was used at level of significance of 5%.

**Results:**

The mean DMFT index was 1.09 and 125 (24.3%) children had orthodontic treatment needs (DAI ≥ 31). There was a similarity and a weak correlation between the scores of the domains of CPQ_11–14_ and AUQUEI (r ranged between −0.006 and 0.0296). In addition, a significant difference was found between the scores of the two instruments according to the socio-economic variables (p < 0.05) and presence of teeth with carious lesions (p < 0.05).

**Conclusions:**

The general and oral health-related quality of life instruments AUQUEI and CPQ_11–14_ were both found to be useful, and significant influence of socio-economic and clinical variables were detected with both instruments.

## Background

The study of quality of life in populations has become common in recent decades [1,2], motivated by a broader conception of the health and disease process, which takes into account the perception of individuals within the context of their values, expectations, and concerns [3].

Thus, normative clinical evaluation alone has become inadequate to enable professionals to provide the best diagnosis and treatment plan for their patients, because patients’ self-reports with regard to their health outcomes do not always coincide with the clinical evaluation made by professionals [1]. Therefore, it is essential to incorporate the physical, social and psychological variables of patients into clinical management in order to promote the therapeutic process that is best for them [4-8].

To achieve these goals, the aim of several studies has been to evaluate the health-related quality of life (HRQoL) in a generic manner, using the World Health Organization Group of Quality of Life questionnaires [1,3,9,10].

As regards measurement of the perception of health-related quality of life in children and adolescents, several instruments have been developed. There are generic instruments that evaluate measures of quality of life in general, with no link to a specific disease, and other instruments related to specific conditions [11-14]. The generic HRQoL instruments are focused on general living conditions. On the other hand, the specific instruments target certain health condition and are able to detect special situations, for example, the impact of oral diseases on the quality of life of children and adolescents [15].

Among the generic HRQoL questionnaires for children and adolescent, there is the Autoquestionnaire Qualité de Vie Enfant image (AUQUEI), a quality of life scale developed in France by Manificat and Dazord [11] that evaluates the subjective perception of quality of life of children and adolescents from 4 to 12 years-old. It has been translated and validated for the Brazilian Portuguese language by Assumpção Jr. et al. [16]. The AUQUEI instrument evaluates satisfaction, from the child’s point of view, associated with various domains of life and consists of 26 questions related to family and social relationships, leisure, autonomy, among others. It is considered a complete tool for evaluating aspects related to quality of life defined in theoretical models [11,15,17] but has rarely been used in the literature up to date. However, given the growing interest of public health managers and professionals in assessing the quality of life of children and adolescents for planning medical interventions, it is increasingly necessary to test and define the possibilities and advantages of using these instruments for this purpose. In addition, Solans et al. [17] have emphasized the importance of the use of generic and specific questionnaires to assess the conditions of quality of life of children and adolescents in clinical practice and the need to investigate the psychometric adequacy of the instrument.

Therefore, in view of the inseparable association between oral health and systemic health, we must consider that the oral health status of children and adolescents can have great impact on their quality of life as a whole [15,17]. Thus, specific and generic measures could be used as tools to assess the impact of oral conditions on the quality of life of this population [18]. Given the peculiar advantages and disadvantages of each of these instruments, it is important to evaluate the relationship between self-reports presented in response to a specific health-related quality of life instrument (i.e. oral health conditions) and a generic instrument.

In the field of oral health, specific instruments have been developed to evaluate the impact of clinical factors and social determinants of health in oral health-related quality of life [19-21].

Among them, there is the Child Perception Questionnaire instrument (CPQ_11–14_) developed by a group of Canadian researchers, with the purpose of assessing the oral health-related quality of life (OHRQoL) in children and adolescents between 11–14 years of age, and measures their OHRQoL in four domains: oral symptoms, functional limitations, emotional wellbeing and social welfare [19,22-27].

In order to better understand the impact that certain oral conditions cause on the overall quality of life, some researchers have evaluated associations between the results of specific with generic health-related quality of life (HRQoL) instruments [18,28-32].

However, there are very few published studies that have investigated these associations, and to our knowledge, so far no study comparing the results of the CPQ_11–14_ (OHRQoL) and AUQUEI (HRQoL) instruments has been published. Therefore, although the psychometric properties of both questionnaires have previously been tested and validated in a Brazilian population [16,33], the objective of this study was to investigate whether there is convergent validity between the two instruments.

In the literature, it is clear that the social determinants of health influence the disease process, health of populations and their subjective perceptions of OHRQoL and HRQoL [21,26,27,34].

Therefore, the aims of this study were: 1) to test the convergent validity between the domains of AUQUEI and CPQ_11–14_; 2) to assess the difference between the socio-economic, home environmental and clinical variables associated with these instruments.

## Methods

### Ethical aspects

The research Project was submitted to the Research Ethics Committee of the Piracicaba Dental School, University of Campinas, Brazil, and approved under Protocol No. 055/2009. The written informed consent of parents/guardians was obtained.

### Subjects

This was a cross-sectional study with cluster sampling in a representative subsample of the adolescent population of the city of Juiz de Fora, Minas Gerais, Brazil. To calculate the probability of error, a 95% confidence interval level was adopted, 20% accuracy and design effect (deff) of 2. The sample size calculation was based on the DMFT(2.3) and standard deviation (2.72) of an epidemiological survey previously conducted. In addition, the calculation to estimate the sample size was based on the effect of socio-economic and home environmental and clinical characteristics of the OHRQoL, considering a power of 80%, confidence level of 95% and a prevalence ratio to be detected of at least 1.5.

Thus, 12-year-old schoolchildren attending 22 public and private schools were selected according in the conglomerate analysis, based on a random multistage sampling design. First, schools were randomly selected, and in each school schoolchildren who fulfilled the inclusion criteria were included in the sample. A total of 515 schoolchildren, considered representative of the city, were evaluated. Details related to sample calculation have been presented in previous studies [26,27].

### Outcome measures

The schoolchildren were clinically examined at school by two calibrated examiners, in an outdoor setting, under natural light. Community Periodontal Index (CPI) probes (ball-point) and intraoral mirrors were used, in accordance with the World Health Organization recommendations for epidemiological surveys [35].

For the evaluation of caries experience, the DMFT/dmft indices (number of decayed, missing and filled permanent and deciduous teeth) were used and for assessing the need for orthodontic treatment, the DAI index (Dental Aesthetic Index) was used in accordance with the WHO criteria [35]. Before the survey, there was a calibration stage for all clinical variables, performed by a gold standard examiner and good intra-examiner reproducibility (Kappa > 0.91) was reached. The calibration process for data collection is available in Paula et al. [27].

One examiner evaluated the children’s caries experience by means of the DMFT index while the second examiner collected data related to the DAI index.

For the purposes of statistical data analyses, we used component D of the DMFT index, which was dichotomized into absence of carious lesions (D = 0) and presence of caries (D > 0). In addition, the DAI index scores were categorized according to Estioko et al. [36] into ‘without orthodontic treatment need’ (DAI <31) and ‘in need of orthodontic treatment (DAI ≥ 31).

To obtain the socio-economic data, a questionnaire containing questions about family income and the mother’s education was sent to the children’s parents. After the clinical examination, in the school environment, the schoolchildren filled in another questionnaire about family environment, such as household overcrowding, number of siblings and with whom the children live (with both biological parents or not) [27].

The application of Autoquestionnaire Qualité de Vie Enfant Imagé (AUQUEI) followed the methodology proposed by the authors [33] and the schoolchildren were asked to tick off the answer that corresponded to their feelings against the 4 proposed domains in the questionnaire. The questionnaire consisted of 26 questions including the domain of autonomy (independence issues, relationships with peers), leisure (questions related to holidays, birthday and relationship with grandparents), functions (questions related to activity in school, meals, bedtime, going to the doctor.) and family (questions as regards parental figures and herself/himself). The domains were scored individually according to values in a Likert scale: 0 (very sad), 1 (sad), 2 (Happy) and 3 (very happy) and total scores range from 0 to 78 - the lower the value, the worse the quality of life. The AUQUEI was applied to the schoolchildren by a single researcher in the school environment.

The Child Perception Questionnaire (CPQ_11–14_) is an instrument used for the specific evaluation of OHRQoL and has been translated and validated for the Brazilian Portuguese language by Barbosa et al. [33]. The instrument consists of 35 questions divided into four domains: oral symptoms, functional limitations, emotional well-being and welfare. Scores are attributed on a Likert scale, 0–4 (based on the number of points in the scale: “Never” = 0; “Once or twice” = 1; “Sometimes” = 2; “Often” = 3; and “Very often” = 4) so that the score of the entire questionnaire may total from 0–140 points, and higher scores mean worse OHRQoL. The questionnaire was applied in the school environment and answered by the children themselves, according to the methodology of Ramos-Jorge et al. [37].

### Data analysis

Descriptive statistics were used to determine the measures of central tendency and dispersion of the results of the questionnaires. Furthermore, the relative frequency of schoolchildren with no influence on their quality of life was calculated for both instruments.

In order to develop a first comparison between the results of AUQUEI and CPQ_11–14_ we made a division of the sample into 4 groups:G1 = good HRQoL (AUQUEI) and OHRQOL (CPQ_11–14_) reported; G2 = good HRQoL reported and bad OHRQOL; G3 = both bad generic HRQoL and OHRQOL reported; G4 = bad generic HRQoL reported and good OHRQoL. This categorization was based in the concept of the Importance-Performance Analysis (IPA) method with the aim of dividing the sample into groups, in which HQoL and OHRQoL showed similar results (both good or bad) [38] .

The convergence validity between the scores (total and by domain) of the two instruments applied was evaluated by means of the Spearman correlation, which is considered a nonparametric test in order to determine the degree of correlation between two measured variables at ordinal level and arranged in ordered positions in two series. It is considered that r values differing from zero represent the correlation between scores.

As the instruments investigated in this study have inverse scales (higher values of AUQUEI scores represent better health-related quality of life, while higher values of CPQ_11–14_ scores represent poorer oral health-related quality of life), for analysis we followed the recommendation given in the study of de Quadros Coelho et al. [39]. This evaluates the correlations between two instruments for measuring quality of life (WHOQOL-HIV BREF and OHIP-14) presenting inverse score scales. According to de Quadros Coelho et al. [39], *to assess the strength of the correlation, the signs of the coefficients need not be evaluated. The signs show if the variables change in the same direction or in the opposite direction.*

For comparison between the summarized scores of each questionnaire (AUQUEI and CPQ_11–14_) with regard to socio-environmental and clinical variables, the median was calculated and the nonparametric Mann–Whitney test was used to determine statistically significant differences between the categories between the questionnaires.

The statistical package SPSS 15.0 (SPSS Inc., Chicago, IL, USA) software program was used for analysis and a p-value < 0.05 was regarded as being statistically significant.

## Results

Among the 515 schoolchildren participating, 363 (70.5%) were enrolled in public schools; 152 (29.5%) in private schools, and 290 (56.3%) of the children were girls. The mean DMFT index was 1.09 (SD 1.70) and mean dmft index was 0.85 (SD 1.42). Among participants, 85 (16.5%) presented teeth with caries lesions. DAI scores ranged from 14.98 to 56.46 with a mean of 26.04 (SD 6.48) and 125 (24.3%) children had orthodontic treatment needs (DAI ≥ 31).

According to the descriptive data presented in Table 1, the mean total score of AUQUEI instrument was 54 and ranged from 8 to 76. None of the participants reported the condition of “very happy” in all 26 questions of AUQUEI, indicating that all participants showed changes in some quality of life domains proposed by the instrument. With regard to the OHRQoL instrument (CPQ_11–14_) the mean of total score was 23, ranging from 0 to 106, and 3.3% (17) of the schoolchildren marked the option “never” to all questions of the instrument, indicating that they did not have any functional or wellness change related to oral health in any domain of the CPQ_11–14_ instrument.

**Table 1:** **Descriptive statistics for AUQUEI and CPQ**_**11–14**_ **scores**

**Measures**	**AUQUEI** ^**1**^	**CPQ**_**11-14**_^**2**^
Mean	54.03	23.24
SD	9.14	21.94
Median	55	16
Range	8-76	0-106
Absence of impact	0% with score 78	3.3% with score 0

^1^smaller scores means worse generic quality of life, range from 0 to 78.

^2^higher scores means worse specific quality of life (oral health related), range from 0 to106.

Table 2 shows the division of the sample into groups according to the results of CPQ_11–14_ and AUQUEI. It was observed that 39.03% of the sample in G1 group - reported good perception for both overall quality of life (AUQUEI) and oral health-related quality of life (CPQ_11–14_) and 22.52% of schoolchildren reported poor quality of life with both instruments (G3). In contrast, 38.25% of schoolchildren presented differences in the results of quality of life between the generic and specific questionnaire (G2 + G4).

**Table 2:** **Absolute and relative frequency categories of associations between the two quality of life instruments used: HRQoL– AUQUEI and OHRQoL– CPQ**_**11–14**_

**GROUPS**		**n**	**%**
G1	HRQoL good	201	39.03%
	OHRQoL good		
G2	HRQoL good	81	15.73%
	OHRQoL bad		
G3	HRQoL bad	117	22.72%
	OHRQoL bad		
G4	HRQoL bad	116	22.52%
	OHRQoL good		
	**Total**	**515**	**100.00%**

Table 3 presents the results of the correlation between the domains and overall scores of AUQUEI and CPQ_11–14_ questionnaires. We found negative correlations for almost all domain scores of the questionnaires, except for the Leisure domain of the AUQUEI instrument, which did not present statistically significant correlations with the Functional Limitations, Emotional Wellbeing and Social Welfare domains of CPQ_11–14_ and their overall scores.

**Table 3:** **Spearman’s correlation coefficients between the AUQUEI and CPQ**_**11-14**_ **instruments (n = 515)**

		**Domains CPQ**_**11–14**_				
		**Oral symptoms**	**Functional limitations**	**Emotional well-being**	**Social well-being**	**Total CPQ**_**11–14**_
DomainsAUQUEI	Autonomy	- 0.232**	- 0.225**	−0.258**	−0.244**	−0.266**
	Leisure	- 0.110*	−0.045^ns^	−0.006^ns^	−0.074^ns^	−0.066^ns^
	Functions	- 0.235**	- 0.273**	- 0.271**	- 0.275**	- 0.296**
	Family	- 0.190**	- 0.133**	- 0.093*	- 0.117**	- 0.144**
Total AUQUEI		- 0.266**	- 0.251**	- 0.244**	- 0.256**	- 0.288**

*p-value < 0.05.

**p-value < 0.01.

^ns^not statistically significant.

Table 4 presents the comparison of the scores of AUQUEI and CPQ_11–14_ as regards the socio-economic, demographic and clinical characteristics of the sample. With regard to AUQUEI, no significant differences were observed between genders and among schoolchildren with and without orthodontic treatment (p > 0.05). In contrast, for the CPQ_11–14_ questionnaire, we observed statistically significant differences in the perception of quality of life related to oral health of adolescents, associated with all independent variables.

**Table 4:** **Difference between the scores of AUQUEI e CPQ**_11-14_ **for clinical and socio-environmental aspects**

		**TOTAL**	**AUQUEI**		**CPQ**_**11–14**_	
			**Median**	p-value*	**Median**	p-value*
Gender	Female	290	55	p = 0.6649	18	p = 0.04
	Male	225	54		13	
School type	Public	363	53	p < 0.0001	23	p < 0.0001
	Private	152	56		6	
Children lives with both biological parents	No	193	52	p = 0.0003	22	p < 0.0001
	Yes	322	56		12	
Household overcrowding	More 1person/room	76	51	p = 0.0031	25	p < 0.0001
	≤1person/room	439	55		15	
Number of siblings	2or more	259	53	p = 0.0037	20	p < 0.0001
	≤2	256	56		10	
Monthly Family income^#^	≤4minimum wages	239	55	p = 0.0008	21	p < 0.0001
	>4 minimum wages	44	59		4	
Mother’s education	≤8 years	141	54	p = 0.0017	24	p < 0.0001
	>8 years	142	56		12	
Presence of caries lesion	Yes	85	50	p < 0.0001	21	p = 0.0334
	No	430	55		15	
Orthodontic treatment need	Yes	125	56	p = 0.0736	23	p < 0.0001
	No	390	54		14	

*Mann–Whitney, nonparametric test for scores comparison.

^#^Minimum wage at the time of data collection, approximately US$ 290.00.

Thus, in the analysis performed for each variable individually, we observed that children from public schools, females, who did not live with their biological parents; whose household overcrowding exceeded one person per room; who had more than two siblings; whose family income was less than 4 minimum wages; whose mother had less than eight years of schooling; and children who had caries and orthodontic treatment needs, presented the worst CPQ_11–14_ values.

With reference to the clinical data, it was observed that the AUQUEI median scores for children with caries was 50 and for those without caries, 55. Taking into account that for AUQUEI the lower the score values, the worse the self-reported quality of life, the results of the general health-related quality of life instrument (AUQUEI) were shown to differ statistically between children with presence and absence of carious lesions (p < 0.0001). Similarly, it was noted that the median scores of the oral health-related quality of life instrument (CPQ_11–14_) in schoolchildren with caries was 21, and for those without caries it was 15.5. Taking into account that for CPQ_11–14_ the higher the value, the worse the self-reported quality of life, we observed that the results of OHRQoL were statistically different for children with the presence and absence of caries lesions (p <0.05). Therefore, the presence of caries was associated with a worse self-perception of both general HRQoL and OHRQoL.

As regards the results on the need for orthodontic treatment, defined by DAI index, it was observed that there was no statistically significant difference between the scores of AUQUEI of schoolchildren with and without orthodontic treatment needs (p = 0.0763). On the other hand, this difference was statistically significant (p <0.0001) with regard to the values of CPQ_11–14._

## Discussion

To our knowledge, this is the first study that has made comparisons between the characteristics of the AUQUEI and CPQ_11–14_ instruments. It is also the first time that social and environmental variables associated with a generic and a specific questionnaire have been compared.

The consistency between the results of AUQUEI and CPQ_11–14_ could be verified by the percentage of schoolchildren whose reports were good for both instruments, or conversely, whose reports were also considered bad for both. As shown in Table 2, we found that 61.75% of them showed similarity in the interpretation of the AUQUEI and CPQ_11–14_ answers. This same convergence of results was also observed for the analysis shown in Table 4. By means of the Spearman correlation, convergent validity values were found between almost all of the domains of AUQUEI and CPQ_11–14_.

The methodology of interpretation of associations using positive and negative correlation to compare specific and generic quality of life questionnaires in cases in which the instruments presented inverse scales, by using the Spearman correlation test, has also been used in other studies, such as Santos et al. [30] and de Quadros Coelho et al. [39]. However, since this is the first study that evaluated the correlation between the results of CPQ_11–14_ and AUQUEI instruments, it is not possible to draw direct comparisons with pre-existing studies in the literature.

Nevertheless, the few studies that have evaluated the correlation between generic HRQoL with specific OHRQoL instruments have also found values close to those of the present study. In the study by Santos et al. [30] comparing the WHOQOL-Bref and the OHIP-14, correlations ranging from −0.1 to −0.2 were found. The study of de Quadros Coelho et al. [39] found correlation ranging from −0.107 to −0.3. In the present study the correlation ranged from 0.0 to −0.2. Considering that there is perfect negative correlation with values of −1 and perfect positive correlation with +1, the correlations closer to zero are considered weaker. In the present study and in similar articles found in the literature, using the same methodology of analysis, a statistically significant, but weak correlation was observed between the instruments (ranging from −0.006 to - 0.296, mean of −0.1943). Therefore, our findings corroborate the hypothesis of the aforementioned authors that these instruments measure different domains of quality of life with distinct constructs. However, it is necessary the application of these instruments in populations with other socio-economic status, cultures and dental status in order to support or refute the evidence found here.

The results of this study revealed that the social determinants of health, including socio-economic and environmental factors were strongly associated with the subjective perceptions of schoolchildren, whether they were related to the results of CPQ_11–14_ or AUQUEI. It was clear that subjective perceptions of quality of life (generic or specific) were associated with the social, environmental, cultural and political context of each individual [27,40,41].

With respect to the clinical variables, we found that dental caries experience was strongly associated with a worse perception of overall quality of life, as measured by AUQUEI, and as can be seen in the proportion between groups and the results of the nonparametric test (Table 4). These findings corroborate those reported by Ribeiro et al. [42] who found that severe caries in preschoolers impaired their overall quality of life, which was measured by the AUQUEI instrument, unlike caries-free children. However, to our knowledge, this is the first study to assess the difference in oral health on overall quality of life measured by the instrument AUQUEI in schoolchildren aged 12 years.

Easton et al. [43] also used a generic quality of life questionnaire (Toddler Child Quality of Life Questionnaire – ITQOL) and found that caries-free preschool children showed better quality of life reports compared with those who had acute or chronic caries with pain. In addition, the study of Fontanive et al. [44],in which adults and elderly persons answered the WHOQOL-Bref questionnaire, one of the most important generic quality of life questionnaires used by researchers, reported the association of caries and the need for prostheses with quality of life. Thus, our results provide important information on the influence of dental caries on overall quality of life of schoolchildren, confirming the findings of Vazquez et al. [45] whose study found an association between oral conditions and WHOQOL-Bref.

With regard to the oral health related quality of life instrument, the differences observed in the results of CPQ_11–14_ scores were also statistically significant for the absence versus presence of caries. This finding is in agreement with numerous other published studies that found associations between oral health and OHRQoL [22-27,45] and highlights the influence of oral health on daily activities of children and adolescents and the importance of these measures for clinical practice.

Furthermore, considering the clinical variables, the results of application of the CPQ_11–14_ instrument showed statistically significant associations between the perceptions of schoolchildren about the influence of their conditions of malocclusion on OHRQoL. Other studies have also found associations between these variables, such as those of Zhang et al. [46], Locker et al. [25] and Paula et al. [27]. Bernabé et al. [28] highlighted the ability of OHRQoL instruments to detect the impact of conditions of malocclusionon the lives of adolescents and found that those with normative need for orthodontic treatment (DAI index) reported the worst OHRQoL.

On the other hand, there were no statistically significant differences between the scores of AUQUEI for participants with and without orthodontic treatment needs. One hypothesis for this finding is that the goals of the AUQUEI and CPQ_11–14_ questionnaires are different, and so are their questions and domains. This would make it difficult for AUQUEI to adequately measure subjective perceptions related to dental aesthetics comprised by the DAI index, contrary to that which occurs with carious lesions, which are more likely to generate pain and discomfort, and consequently have a greater influence on quality of life. Liu et al. [47] presented a review of the literature on the subject and concluded that there was association between malocclusion/treatment needs and quality of life (by means of ageneric or specific questionnaire), but it was weak. The authors also emphasized that the result of this association may be influenced by the type of questionnaire adopted. In this regard, Locker et al. [25] reaffirmed the need for a specific instrument, such as CPQ_11–14_ for a more accurate evaluation of the different perceptions of orthodontic conditions, and in turn, emphasized the need for further studies on the usefulness of these instruments. This fact must be taken into consideration by researchers and clinicians when selecting a generic quality of life tool to assess the impact of a specific disease on HRQoL, because the association will be not always found [13].

To date, only one study has investigated the association between the results of the CPQ_11–14_ and AUQUEI to evaluate the quality of life of its participants [48]. The aim of the mentioned study was to assess the general and specific oral health related quality of life of HIV-infected children. However, the authors did not investigate the difference in social and environmental aspects as confounders in the model of association between OHRQoL and HRQoL, as was done in the present study. In the abovementioned study, the authors observed that there was an association between the condition of being HIV positive and the subjects’ general and specific OHRQoL measured by means of the AUQUEI and CPQ_11–14_ instruments.

Other studies that have investigated the associations between generic and specific OHRQoL instruments, such as Fontanive et al. [44], who investigated associations between clinical oral variables and the WHOQOL, and Santos et al. [30] who compared two generic measures (short form CPQ_11–14_ and WHOQOL-Bref) also observed the same associations.

As shown in Table 4, it was verified that socio-economic and family aspects presented a strong association with general and oral health-related quality of life. Despite the lack of studies comparing the results of AUQUEI scores in different social and environmental conditions, the association between quality of life and social determinants of health has been extensively studied in the scientific literature and should be taken into account when formulating any public health policy.

Based on the differences and similarities of the results found for the measures evaluated, we concluded that both questionnaires are useful and important in order to implement holistic strategies for oral health promotion based on a sociodental approach [4,6]. Moreover, irrespective of the quality of life questionnaire applied, aspects related to the social determinants of health should be observed, since the present study makes clear the influence of these factors on the results measured by the two types of instruments.

The results of the present study should be considered within some limitations, such as the low prevalence of oral diseases, which may have influenced the strength of the association found. In addition, we did not evaluate the presence of general diseases or health problems that could have influenced the results of AUQUEI, and the cross-sectional study design did not allow us to assess a dynamic relationship of cause and effect over time between independent variables and the results of AUQUEI and CPQ_11–14_.

## Conclusions

In conclusion, the generic (AUQUEI) and the specific oral health-related (CPQ_11–14_) quality of life instruments showed correlation, with weak association, and the analysis of socio-economic and home environmental and clinical variables showed association when measured with both instruments.

### Supporting data

The authors declare that they have no supporting data for this study.

## Abbreviations

AUQUEI: Autoquestionnaire Qualité de Vie Enfant image (AUQUEI)
CPQ_11–14_: Child Perception Questionnaire
DMFT and dmft index: Number of decayed, missing and filled permanent and deciduous teeth
DAI index: Dental Aesthetic Index
HRQoL: Health-related quality of life
OHRQoL: Oral health-related quality of life
